# Treatment Strategies for Residual Disease following Neoadjuvant Chemotherapy in Patients with Early-Stage Breast Cancer

**DOI:** 10.3390/curroncol29080458

**Published:** 2022-08-16

**Authors:** Hikmat Abdel-Razeq, Hanan Khalil, Hazem I. Assi, Tarek Bou Dargham

**Affiliations:** 1 Department of Internal Medicine, King Hussein Cancer Center, Amman 11941, Jordan; 2 School of Medicine, University of Jordan, Amman 11941, Jordan; 3 Department of Internal Medicine, Naef K. Basile Cancer Institute, American University of Beirut Medical Center, Beirut 1107, Lebanon; 4 School of Medicine, University of Balamand, Koura 3843, Lebanon

**Keywords:** breast cancer, HER2-positive, HER2-negative, pathologic complete response, post-neoadjuvant treatment, residual disease

## Abstract

Breast cancer continues to be the most diagnosed cancer among women worldwide. Neoadjuvant chemotherapy is the standard of care for breast cancer patients with locally advanced disease and patients with poor pathological features, such as triple-negative (TN) or human epidermal growth factor receptor-2 (HER2)-positive subtypes. Neoadjuvant therapy offers several advantages, including better surgical outcomes, early systemic treatment for micro-metastases, and accurate tumor biology and chemosensitivity assessment. Multiple studies have shown that achieving pathological complete response (pCR) following neoadjuvant chemotherapy is associated with better prognosis and better treatment outcomes; almost half of such patients may fail to achieve pCR. Tumor proliferative index, hormone receptor (HR) status, and HER2 expression are the major predictors of pCR. Strategies to improve pCR have been dependent on augmenting neoadjuvant chemotherapy with the addition of taxanes and dual anti-HER2 targeted therapy in patients with HER2-positive tumor, and more recently, immunotherapy for patients with TN disease. The clinical management of patients with residual disease following neoadjuvant chemotherapy varies and depends mostly on the level of HR expression and HER2 status. Recent data have suggested that switching trastuzumab to trastuzumab-emtansine (T-DM1) in patients with HER2-positive disease and the addition of capecitabine for patients with HER2-negative and HR-negative subtype is associated with a better outcome; both strategies are incorporated into current clinical practice guidelines. This paper reviews available and ongoing studies addressing strategies to better manage patients who continue to have residual disease following neoadjuvant chemotherapy.

## 1. Introduction

Breast cancer is the most commonly diagnosed cancer among women worldwide and is considered a leading cause of cancer-related mortality [1,2]. Neoadjuvant therapy for breast cancer has long been considered to offer patients an early treatment for their tumors, especially for those who present with locally-advanced inoperable cancers, providing the benefit from downstaging the tumor and regional lymph nodes and improving the surgical outcomes by increasing the rate of breast-conserving surgery (BCS) [3]. Neoadjuvant therapy can help delay surgery for those awaiting genetic testing results or the resolution of an intercurrent illness and pregnancy. Additionally, neoadjuvant therapy allows the accurate assessment of tumor biology and chemosensitivity. It offers early systemic treatment for micro-metastases. It gives us the opportunity to modify adjuvant treatment according to pathology results, especially for high-risk patients, such as those with TN or HER2-positive disease [4]. Neoadjuvant therapy can also benefit patients with limited axillary nodal involvement (N1): such patients may avoid axillary lymph node dissection if converted to node-negative disease with neoadjuvant therapy. In such cases, sentinel lymph node sampling can be an acceptable alternative to full axillary dissection with its associated morbidity.

Despite the introduction of many targeted drugs and the utilization of immunotherapy, a relatively high percentage of patients fail to achieve pCR. Following neoadjuvant chemotherapy, patients with residual disease are at higher risk for local and systemic relapse [5,6]. While patients with HR-positive tumors receive adjuvant hormonal therapy, with or without radiation therapy, patients with TN disease may not receive any post-surgical systemic treatment.

This paper reviews available and ongoing studies addressing strategies to better manage patients who continue to have residual disease following neoadjuvant chemotherapy. We first describe pCR, which is the standard clinical measure of treatment outcome for breast cancer. This is followed by a review of the management of residual HER2-positive disease, the management of residual HER2-negative disease, immunotherapy, and PARP inhibitors in *BRCA*-positive patients.

## 2. Pathological Complete Response (pCR)

The definition of pCR varies across many clinical trials, but the most widely accepted definition includes an absence of residual invasive disease in the breast and axillary nodes (ypT0/is, ypN0). The most stringent definition, however, is the one that mandates no residual invasive or non-invasive (in situ) disease in both the breast and axillary lymph nodes (ypT0, ypN0) [7,8,9]. However, the presence of non-invasive carcinoma seems to have no negative impact on the long-term outcome of breast cancer patients [5].

Many studies have confirmed the association between pCR and event-free survival (EFS) and overall survival (OS), especially in patients with HR-negative, TN, or HER2-positive disease. Even from early neoadjuvant trials, such an association was very apparent [10]. Although the National Surgical Adjuvant Breast and Bowel Project (NSABP) B-18 trial failed to demonstrate relapse-free survival (RFS) or OS advantages of neoadjuvant chemotherapy compared to the adjuvant one, it did show that patients who achieved pCR had better disease-free survival (DFS) (hazard ratio, 0.47; *p* = 0.0001) and OS (hazard ratio, 0.32; *p* = 0.0001) compared to those with residual disease [10]. Given the high correlation of pCR and clinical outcomes, both the European Medicines Agency (EMA) and the U.S. Food and Drug Administration (FDA) endorsed the use of pCR as an endpoint for the assessment of neoadjuvant treatment for HER2-positive and TN disease breast cancer patients [11,12].

The Collaborative Trials in Neoadjuvant Breast Cancer (CTNeoBC) working group collected data from 12 international trials with almost 12,000 patients enrolled. Eradication of tumor from both the axillary lymph nodes and the breast (ypT0, ypN0 or ypT0/is, ypN0) was associated with better EFS and OS. This association was strongest among patients with HER2-positive/HR-negative tumors treated with trastuzumab and among those with TN disease [5]. Another meta-analysis that included 36 studies with 5768 patients with HER2-positive disease concluded that patients who achieved pCR, compared to those with residual disease, had better EFS (hazard ratio, 0.37; 95% probability interval (PI), 0.32–0.43). The association was more noticeable in patients with HR-negative disease (hazard ratio, 0.29; 95% PI, 0.24–0.36) than in those with HR-positive disease (hazard ratio, 0.52; 95% PI, 0.40–0.66) [13]. A more recent and more extensive meta-analysis of data involving 52 studies that enrolled 27,895 patients with breast cancer confirmed that the higher the pCR following neoadjuvant therapy, the lower the risk of disease recurrence (hazard ratio, 0.31; 95% CI, 0.24–0.39) and death (hazard ratio, 0.22; 95% CI, 0.15–0.30) [14].

## 3. Management of Residual HER2-Positive Disease

### 3.1. Current Neoadjuvant Standards

Almost 20% of all breast cancers express HER2 [15,16]. This subtype of tumors is characterized by the amplification of the HER2 gene that encodes for a type of tyrosine kinase receptor among other types of genes within the same family: HER1 (sometimes referred to as EGFR), HER2, HER3, and HER4. Activation of these receptors results in the activation of downstream intracellular signaling pathways, resulting in continued cellular proliferation and survival. Targeting this oncogenic driver will offer a therapeutic advantage to tumors harboring it [17,18,19].

HER2-positive tumors tend to have more aggressive behavior and usually have worse outcomes than other subtypes [20]. Such worse outcomes were relatively offset since the introduction of anti-HER2 therapy, which improved the prognosis of breast cancer patients in both advanced and early disease settings [21].

In addition to adjuvant chemotherapy, trastuzumab for 1 year is the current standard of care for HER2-positive early-stage breast cancer patients. The benefit of adding trastuzumab to chemotherapy was proven in many large randomized trials: HERA [22,23,24,25], NSABP-B31, NCCTG N9831 [26,27,28], BCIRG 006 [29], and FNCLCC-PACS 04 trials [30]. Data from these trials, except the FNCLCC-PACS 04 [30], showed that trastuzumab when added to chemotherapy was associated with significantly better DFS and OS compared to chemotherapy alone, a benefit which was maintained with longer follow-up [25,29].

In an attempt to further improve the DFS of this special subtype of breast cancer patients, investigators studied an extended adjuvant treatment with neratinib, a pan-HER2 inhibitor, for one year after completing a full year of trastuzumab in the ExteNET trial. Patients (n = 2840) with HER2-positive early breast cancer were randomized to receive either neratinib or placebo. Patients in the neratinib group fared better in terms of 5-year DFS: 90.2% compared to 87.7% in the placebo group (hazard ratio, 0.73; 95% CI, 0.57–0.92; *p* = 0.008) [31]. The study was updated recently, and neratinib was associated with better OS at 8 years (hazard ratio, 0.79; 95% CI, 0.55–1.13). Additionally, central nervous system (CNS) events were fewer with neratinib [32].

The APHINITY trial evaluated the benefit of adding another anti-HER2 drug (pertuzumab) to a combination of both trastuzumab and chemotherapy in the adjuvant settings [33]. The results of this trial were not outstanding as was expected, since the addition of pertuzumab conferred only a small benefit in terms of 3-year invasive disease-free survival (iDFS): 94.1% versus 93.2% (hazard ratio, 0.81; 95% CI, 0.66–1.00; *p* = 0.045). Patients in the high-risk category (with node-positive disease) had the greatest benefit: a 3-year iDFS of 92.0% versus 90.2% (hazard ratio, 0.77; 95% CI, 0.62–0.96; *p* = 0.02) [33].

Taxane-based regimens, with or without anthracycline, plus dual HER2 blockade with pertuzumab and trastuzumab, remain the current standard of care for neoadjuvant treatment for patients with HER2-positive disease [34,35]. Over the years, many trials have involved different chemotherapy and anti-HER2 combinations with varying rates of pCR as high as 70% [36,37], as shown in Table 1.

The finding of residual disease after neoadjuvant treatment indicates chemotherapy-resistant tissues in the tumor [7,42]. Chemotherapy modification during neoadjuvant therapy, based on the failure of clinical response according to the physical examination, with or without imaging studies, showed no improvement in pCR, as was demonstrated in several studies, including the GeparTrio trial [43,44,45].

Over the years, multiple strategies have been tried to improve pCR rates and recurrence-free and long-term survivals. Such strategies involved the addition of new drugs in the neoadjuvant settings, extending the duration of neoadjuvant therapy, dose-intensification, and trying to use concurrent chemoradiation regimens; all failed to show significant survival differences [46,47,48,49,50,51].

### 3.2. Post Neoadjuvant Treatment

Another approach, termed the post-neoadjuvant treatment, offers additional adjuvant treatment for patients who do not achieve pCR after neoadjuvant therapy to overcome tumor resistance. The concept of post-neoadjuvant therapy has been studied with frequent failures or statistically insignificant results [52,53,54,55,56].

Chemotherapies have a narrow therapeutic index because they are tumor non-selective, causing multiple systemic toxicities. Antibody-drug conjugates (ADCs) are drugs that link a tumor-specific antibody to a cytotoxic drug. ADCs are designed to limit collateral damage to normal cells by specifically delivering cytotoxic agents to tumor cells, thus improving their therapeutic index.

Trastuzumab-emtansine (T-DM1) is an example of these antibody-drug conjugates, consisting of a monoclonal antibody against HER2 combined with a thioether linker and the antimicrotubule chemotherapy maytansine [57]. Its activity was first demonstrated in the metastatic settings in EMILIA and TH3RESA studies [58,59,60,61]. To study the activity of T-DM1 in the post-neoadjuvant settings, a phase III trial (the KATHERINE trial) involved 1486 HER2-positive patients with residual disease in the breast or axilla after neoadjuvant treatment (taxane-based chemotherapy, with or without anthracyclines) plus trastuzumab (with or without pertuzumab) who received either 14 cycles of T-DM1 or 14 cycles of trastuzumab; the primary endpoint was iDFS. Patients included were those with T1-T4 tumors, N0-N3 lymph nodes, and M0 (excluding T1aN0 or T1bN0) at presentation. The residual invasive disease was detected pathologically in the breast or axilla at the time of surgery after neoadjuvant treatment. Hormone receptors were positive in 72.3% of patients, and anthracyclines were given to 76.9%. The majority (80%) of patients received trastuzumab alone, while 18% were treated with dual anti-HER2: both trastuzumab and pertuzumab. Patients with positive margins after breast-conserving surgery, those with gross residual disease after mastectomy, and those who had progressed while taking neoadjuvant therapy were excluded. Additionally, patients with cardiopulmonary compromise, including patients with class II or higher, New York Heart Association (NYHA) heart failure, and patients with a history of deteriorating ejection fraction (EF) to less than 40% with previous therapies were excluded. After a median follow-up of 41.4 months, the 3-year iDFS rates were 88.3% in the T-DM1 group compared to 77% in the trastuzumab group (hazard ratio, 0.50; 95% CI, 0.39–0.64; *p* < 0.001), which represented a 50% reduction in the risk of recurrence of invasive disease or death. The rate of distant recurrences was 10.5% with T-DM1 versus 15.9% with trastuzumab, and all subgroups benefited from T-DM1 [62].

Criticism to the KATHERINE trial was the reliance on the pretreatment core biopsy to determine the HER2 status. There was an apparent loss of HER2 positivity in a subgroup of patients. The KATHERINE trial could not answer whether adjuvant T-DM1 was specifically beneficial in this subgroup. The most common grade 3 or more adverse events observed in this trial were thrombocytopenia and hypertension in the T-DM1 arm and hypertension and radiation-induced skin injury with trastuzumab. Despite the fact that more patients experienced thrombocytopenia in the T-DM1 arm, the percentages of patients with grade 3 or more hemorrhages were similar in both arms (0.4% in the T-DM1 group and 0.3% in the trastuzumab group). Based on this data, T-DM1 is widely accepted as the standard anti-HER2 treatment in patients who fail to achieve pCR after neoadjuvant anti-HER2 therapy.

### 3.3. Ongoing Trials Investigating Other Anti-HER2 Agents in the Post Neoadjuvant Settings

At least two additional clinical trials are ongoing to address residual disease following neoadjuvant therapy in HER2-positive breast cancer patients utilizing tumor vaccines. The first is recruiting patients with residual disease following taxanes and trastuzumab-based neoadjuvant therapy to receive nelipepimut-S, a vaccine derived from the E75 peptide. This antigen is expressed in the extracellular domain of HER2, coupled with granulocyte-macrophage colony-stimulating factor (GM-CSF). Nelipepimut-S will be administered for 2 years in the adjuvant settings with a primary endpoint of DFS [63]. Another randomized phase II trial investigates two dendritic cell vaccines (DC1 and WOKVAC) in HER2-positive patients who fail to achieve pCR after a trastuzumab-containing neoadjuvant therapy. The rationale is that the vaccines may induce an anti-tumor immune response. Patients are randomized to DC1 or WOKVAC, administered for 1 year, with the primary endpoint being DFS [64].

## 4. Management of Residual HER2-Negative Disease

### 4.1. Current Neoadjuvant Standards

The majority of breast cancers do not express HER2. This group can be divided into two subtypes. The first is HR-negative, commonly described as TN, representing 10–15% of all breast cancers. Several trials have attempted to improve pCR in this group of patients but with modest success [65,66]. The classical anthracycline, cyclophosphamide, and taxanes-based regimen resulted in pCR of 30–44%. The addition of carboplatin to this regimen improved pCR to around 55% [67]. Anthracycline-sparing regimens were also tried in patients with TN disease; carboplatin in combination with a taxane (without anthracycline) in regimens given for 12 to 18 weeks resulted in pCR rates ranging from 45% to 55% with a favorable toxicity profile, as shown in Table 2 [68,69].

The other subtype, the most common, is HR-positive disease, which is divided into two categories, luminal A and luminal B (HER2-negative). The difference between these breast cancer subtypes is the level of Ki67 expression, which indicates cell proliferation. These differences allow the classification of luminal A as having a low proliferation rate due to its low expression of Ki67 and luminal B (HER2-negative) as having a higher proliferation rate due to its higher level of Ki67 expression [78,79,80]. Ki67 can be considered an important biomarker in differentiating between subtypes and can serve as a prognostic marker for patients who fail to achieve pCR. In a study that enrolled 435 patients with stage IIB-IIIA disease treated with an anthracycline and taxanes with residual disease following neoadjuvant therapy, changes in Ki67 expression in the original core biopsy and the surgical specimen were evaluated in relation to DFS and OS. Forty-five percent of patients had luminal B-like tumors, 25% had luminal A-like, 14% had TN, 11% had triple-positive, and 5% had HER2-positive disease. Fifty-seven percent of participants had a decline in Ki67 percentage, and this decrease was directly correlated with a better DFS and OS, specifically in luminal B subtypes. However, no difference was observed in DFS or OS patients with luminal A, TN, HER2-positive, and luminal B/HER2-positive subtypes [81].

### 4.2. Residual Disease in HER2-Negative Breast Cancer

Despite the ultimate goal of achieving pCR in breast cancer, a high percentage of patients are left with residual disease post-treatment and surgery. The CREATE-X trial was designed to treat residual diseases in HER2-negative patients who did not achieve pCR. Breast cancer patients with residual disease following neoadjuvant chemotherapy were divided into two groups: one received the standard adjuvant care, and the other received standard adjuvant care plus capecitabine. The study included both hormone receptor-positive and negative patients and used DFS and OS as its endpoints. The study was terminated early, and final analysis showed that patients treated with capecitabine had a better 5-year DFS than the control group (74.1% versus 67.6%; hazard ratio for recurrence, second cancer, or death, 0.70; 95% CI, 0.53–0.92; *p* = 0.01). Overall survival was also longer in the capecitabine group (89.2%) compared to 83.6% in the control group (hazard ratio for death, 0.59; 95% CI, 0.39–0.90; *p* = 0.01). The difference was more significant in patients with TN disease: 5-year DFS was 69.8% in the capecitabine group compared to 56.1% in the control arm (hazard ratio for recurrence, second cancer, or death, 0.58; 95% CI, 0.39–0.87). The OS rate was also better: 78.8% compared to 70.3% (hazard ratio for death, 0.52; 95% CI, 0.30–0.90). Hand–foot syndrome was the most frequent adverse event, occurring in 73.4%, including 11.1% with a grade 3 event among the capecitabine group. Diarrhea, leukopenia, thrombocytopenia, neutropenia, and anemia were also encountered [56].

Additional clinical trials are being conducted to study the role of capecitabine as an adjuvant therapy following neoadjuvant chemotherapy in patients with TN breast cancer. The GEICAM/CIBOMA is a phase III trial that studied the value of adding capecitabine in patients with TN disease regardless of their neoadjuvant chemotherapy outcomes. There were 876 patients: 55.9% were lymph node-negative and 73.9% had a basal phenotype. After a median follow-up of 7.3 years (range: 0.0 to 11.1), DFS was not significantly prolonged with capecitabine compared to the control arm (hazard ratio, 0.82; 95% CI, 0.63 to 1.06; *p* = 0.136). Five-year DFS was 79.6% (95% CI, 75.8% to 83.4%) with capecitabine and 76.8% (95% CI, 72.7% to 80.9%) with observation. No statistical difference was observed in OS, either (hazard ratio, 0.92; 95% CI, 0.66 to 1.28; *p* = 0.623) [82,83].

## 5. Immunotherapy

### 5.1. Immunotherapy in Neoadjuvant Settings

Compared to other solid tumors or hematological malignancies, immunotherapy in breast cancer was delayed. Recently, promising data have started to appear, mostly in patients with the TN subtype [84]. The KEYNOTE-522 was a phase III randomized trial that assessed the use of the programmed cell death protein-1 (PD-1) inhibitor, pembrolizumab, and chemotherapy in the neoadjuvant therapy of patients with TN disease. Patients (n = 602) were randomized to receive chemotherapy (paclitaxel and carboplatin, then cyclophosphamide and anthracycline) plus pembrolizumab or chemotherapy plus placebo. The combination of pembrolizumab and chemotherapy resulted in a significant increase in pCR rates (64.8%; 95% CI, 59.9–69.5) compared to chemotherapy alone (51.2%; 95% CI, 44.1–58.3); the estimated treatment difference was 13.6% (95% CI, 5.4–21.8; *p* < 0.001). After a median follow-up of 15.5 months, 7.4% of patients in the pembrolizumab-chemotherapy group and 11.8% in the placebo-chemotherapy group had an event (hazard ratio, 0.63; 95% CI, 0.43–0.93) [77]. The study was also updated during the last annual meeting of the American Society of Clinical Oncology (ASCO). Compared with placebo, and after a median follow-up of 39.1 months, pembrolizumab added to neoadjuvant chemotherapy was associated with lower residual cancer burden (RCB) in patients with TN disease and with prolonged EFS even in patients who failed to achieve pCR [85].

The addition of atezolizumab to a 24-week carboplatin/nab-paclitaxel chemotherapy backbone was also tried in a phase III trial (NeoTRIP) in patients with locally advanced TN disease. The pCR rates were not significantly different between the two study arms (40.8% with chemotherapy compared to 43.5% with atezolizumab) [86]. Atezolizumab was also tried in the Impassion-031 trial, which randomized 333 previously untreated stage II-III patients with TN disease to atezolizumab combined with neoadjuvant chemotherapy (nab-paclitaxel, doxorubicin, and cyclophosphamide) versus chemotherapy alone. In the PD-L1-positive patients, pCR was reported in 69% in the atezolizumab plus chemotherapy group versus 49% in patients in the placebo plus chemotherapy group (rate difference, 20%; 95% CI, 4–35; *p* = 0.021) [87]. Regardless of PD-L1 expression, atezolizumab increased pCR rates from 41.1% in the chemotherapy-alone group to 57.6% in the combination group (rate difference, 17%; 95% CI, 6–27; *p* = 0.0044).

### 5.2. Immunotherapy for Residual Disease

Given the encouraging results of the upfront use of immunotherapy in the neoadjuvant setting of early-stage breast cancer, it was natural to try these agents in patients with residual disease following neoadjuvant therapy. This concept is being tested in the SWOG 1418, a randomized, open-label, phase III trial that will compare the iDFS of over 1100 patients with TNBC who fail to achieve pCR, with residual invasive breast cancer ≥ 1 cm, and/or positive lymph nodes after neoadjuvant therapy. Patients are randomized to receive one year of pembrolizumab as adjuvant therapy compared to none in the entire study population. The study will stratify patients by nodal stage (ypNo vs. ypN+), residual tumor (≥2 cm vs. <2 cm), PD-L1 status (positive vs. negative), and prior adjuvant chemotherapy [88].

## 6. PARP Inhibitors in BRCA-Positive Patients

Olaparib, a Poly ADP-ribose polymerase (PARP) inhibitor, was also tested in a subgroup of patients with BRCA1 or BRCA2 germline pathogenic or likely pathogenic variants who had residual disease following neoadjuvant chemotherapy or in high-risk patients following adjuvant therapy. The OlympiA is a phase III, double-blind, randomized trial that involved 1836 patients with high-risk, HER2-negative early breast cancer who had received local treatment plus adjuvant or neoadjuvant chemotherapy. Patients were randomized to receive oral olaparib or placebo for one full year. At a median follow-up of 2.5 years (event-driven analysis), the 3-year iDFS was 85.9% in the olaparib group and 77.1% in the placebo group (95% CI, 4.5–13.0; hazard ratio for invasive disease or death: 0.58; 99.5% CI, 0.41–0.82; *p* < 0.001). Similarly, the 3-year DFS was significantly higher in the olaparib group: 87.5% compared to 80.4% in the placebo group (95% CI, 3.0 to 11.1; hazard ratio for distant disease or death, 0.57; 99.5% CI, 0.39 to 0.83; *p* < 0.001) [89]. This study gives patients with BRCA1 or BRCA2 another option to improve treatment outcomes but adds a little confusion in choosing between the many newly introduced options in this setting.

## 7. Conclusions and Future Directions

**Figure 1:**
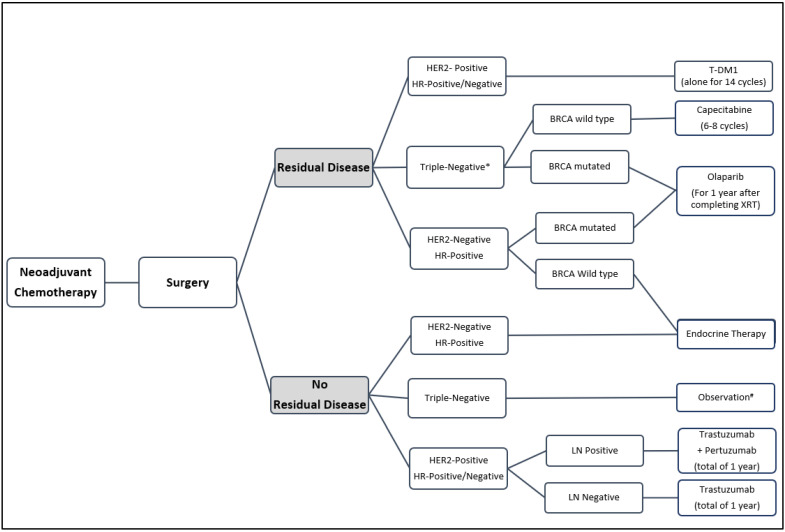
Suggested algorithm for management of residual disease following neoadjuvant therapy and surgery. * Continue immunotherapy if used prior to surgery. # May consider immunotherapy in clinical trials. HER2: human epidermal growth factor receptor 2; HR: hormone receptor; LN: lymph node; XRT: radiation therapy; T-DM1: trastuzumab emtansine.

While significant progress has been made in the neoadjuvant and adjuvant therapy of early-stage breast cancer [90,91,92,93], several questions remain unanswered: What is the impact of adjuvant immunotherapy, specifically for those who fail to achieve pCR? How would capecitabine then be incorporated? What is the role of other targeted agents such as PARP inhibitors? Would the other recently introduced antibody-drug conjugates, such as trastuzumab deruxtecan, be better than T-DM1 in patients with HER2-positive disease [94]? Can we better select patients for more appropriate therapy based on biomarkers? These questions, and many others, will hopefully be answered by many of the ongoing and planned clinical trials. Figure 1 summarizes the current knowledge related to the management of breast cancer post-neoadjuvant therapy.

## Figures and Tables

**Table 1:** Rates of pathologic complete response (pCR) in HER2-positive subtypes.

Clinical Trial	Dual HER2 Therapy	pCR Rate with Single Anti-HER2 Agent (%)	pCR Rate with Dual Anti-HER2 Therapy (%)
NeoALTTO [38]	L + T	30 (95% CI, 22.4–37.5) with T 25 (95% CI, 18.1–32.3) with L	51 (95% CI, 43.1–59.5; *p* = 0.0001)
NeoSphere [39]	T + P	29 (95% CI, 20.6–38.5) with T 24 (95% CI, 15.8–33.7) with P	46 (95% CI, 36.1–55.7; *p* = 0.0141)
CALGB 40601 [40]	L + T	40 (95% CI, 32–49) with T 32 (95% CI, 22–44) with L	51 (95% CI, 42–60; *p* = 0.11)
NSABP B-41 [41]	L + T	53 (95% CI, 44.9–59.5) with T 53 (95% CI, 44.4–60.3) with L	62 (95% CI, 54.3–68.8; *p* = 0.095)
TRYPHAENA [36]	T + P	NA	57–66

HER2: human epidermal growth factor receptor; L: lapatinib; T: trastuzumab; P: pertuzumab; pCR: pathologic complete response; NA: not available.

**Table 2:** Pathologic complete response (pCR) rates using different neoadjuvant therapies for triple-negative (TN) disease.

Treatment	Pathologic Complete Response (pCR) Rates (%)
Anthracycline [70,71]	14–47
Anthracycline + taxane [72]	17–39
Anthracycline + cyclophosphamide followed by taxanes [65,66,73]	30–44
Carboplatin to a backbone of anthracycline/taxanes [65,66,67,68]	52–57
Platinum monotherapy (carboplatin, cisplatin) [74,75]	23–90 *
Gemcitabine + carboplatin + iniparib [76]	36
Pembrolizumab + standard chemotherapy [77]	64.8

* Highest among patients with BRCA gene mutations.
